# The Effect of Perioperative Vitamin C on Postoperative Analgesic Consumption: A Meta-Analysis of Randomized Controlled Trials

**DOI:** 10.3390/nu12103109

**Published:** 2020-10-12

**Authors:** Kuo-Chuan Hung, Yao-Tsung Lin, Kee-Hsin Chen, Li-Kai Wang, Jen-Yin Chen, Ying-Jen Chang, Shao-Chun Wu, Min-Hsien Chiang, Cheuk-Kwan Sun

**Affiliations:** 1 Department of Anesthesiology, Chi Mei Medical Center, Tainan City 71004, Taiwan; ed102605@gmail.com (K.-C.H.); anekevin@hotmail.com (Y.-T.L.); anesth@gmail.com (L.-K.W.); chenjenyin@gmail.com (J.-Y.C.); 0201day@yahoo.com.tw (Y.-J.C.); 2 Department of Health and Nutrition, Chia Nan University of Pharmacy and Science, Tainan City 71710, Taiwan; 3 Center of General Education, Chia Nan University of Pharmacy and Science, Tainan City 71710, Taiwan; 4 Post-Baccalaureate Program in Nursing, College of Nursing, Taipei Medical University, Taipei City 11042, Taiwan; keehsin@gmail.com; 5 Cochrane Taiwan, Taipei Medical University, Taipei City 11042, Taiwan; 6 Center for Nursing and Healthcare Research in Clinical Practice Application, Wan Fang Hospital, Taipei Medical University, Taipei City 11042, Taiwan; 7 Evidence-Based Knowledge Translation Center, Wan Fang Hospital, Taipei Medical University, Taipei City 11042, Taiwan; 8 Department of the Senior Citizen Service Management, Chia Nan University of Pharmacy and Science, Tainan City 71710, Taiwan; 9 College of Health Sciences, Chang Jung Christian University, Tainan City 71101, Taiwan; 10 Department of Anesthesiology, Kaohsiung Chang Gung Memorial Hospital, Chang Gung University College of Medicine, Kaohsiung City 83301, Taiwan; shaochunwu@gmail.com; 11 Department of Emergency Medicine, E-Da Hospital, Kaohsiung City 82445, Taiwan; 12 College of Medicine, I-Shou University, Kaohsiung City 82445, Taiwan

**Keywords:** vitamin C, analgesic requirement, surgery, anesthesia

## Abstract

Because the analgesic effect of vitamin C against acute pain remains poorly addressed, this meta-analysis aimed at investigating its effectiveness against acute postoperative pain. A total of seven randomized controlled trials with placebo/normal controls were identified from PubMed, Cochrane Library, Medline, Google Scholar, and Embase databases. Pooled analysis showed a lower pain score (standardized mean difference (SMD) = −0.68, 95% CI: −1.01 to −0.36, *p* < 0.0001; *I*^2^ = 57%) and a lower morphine consumption (weighted mean difference (WMD) = −2.44 mg, 95% CI: −4.03 to −0.86, *p* = 0.003; *I*^2^ = 52%) in the vitamin group than that in the placebo group within postoperative 1–2 h. At postoperative 24 h, a lower pain score (SMD = −0.65, 95% CI: −1.11 to −0.19, *p* = 0.005; *I*^2^ = 81%) and lower morphine consumption (WMD = −6.74 mg, 95% CI: −9.63 to −3.84, *p* < 0.00001; *I*^2^ = 85%) were also noted in the vitamin group. Subgroup analyses demonstrated significant reductions in pain severity and morphine requirement immediately (1–2 h) and 24 h after surgery for patients receiving intravenous vitamin C but not in the oral subgroup. These findings showed significant reductions in pain score and opioid requirement up to postoperative 24 h, respectively, suggesting the effectiveness of perioperative vitamin C use. Further large-scale trials are warranted to elucidate its optimal intravenous dosage and effectiveness against chronic pain in the postoperative pain control setting.

## 1. Introduction

Postoperative pain, which is present in up to 80% of patients undergoing surgery [1], not only impairs the patients’ quality of life such as sleep quality and level of activity [2] but also contributes to chronic postoperative pain [3]. However, a previous study has shown that less than half of the postoperative patients reported satisfactory pain control [1]. The commonly used postoperative analgesic agents including opioids and non-steroid anti-inflammatory drugs (NSAIDs) are associated with untoward side-effects including respiratory suppression and other relatively minor complications such as nausea or vomiting [4]. In addition, NSAIDs, which are usual adjuvants to opioid-based regimens, are known to contribute to adverse gastrointestinal and nephrotic side-effects. Inadequate pain control is a common tradeoff between optimal postoperative analgesia and drug-associated side-effects [5]. In an attempt to maximize the degree of analgesia while minimizing untoward side-effects, modern practice guidelines recommend a multimodal intervention for postoperative pain control that involves a combination of different analgesics with nonpharmacological approaches [4].

Vitamin C, the L-enantiomer of ascorbate also known as ascorbic acid, is widely known as a water-soluble antioxidant ubiquitously present in a variety of fruits and vegetables. The importance of vitamin C in wound healing and hemostasis was first realized more than 260 years ago when it was found to be a cure for scurvy, a disease characterized by spontaneous bleeding, anemia, and gum ulceration. Besides its role in hemostasis, vitamin C is also known to exhibit analgesic functions. Recent evidence has attributed the antinociceptive action of vitamin C to its antioxidant [6], neuroprotective, and neuromodulatory properties [7,8]. Indeed, vitamin C has been shown to reduce acute pain and the prevalence of complex regional pain syndromes with its antinociceptive effect [9,10,11].

Although the antinociceptive effect of vitamin C against chronic pain has been well documented [10], its effectiveness against acute pain remains poorly addressed. There was only one meta-analytic study attempting to investigate the effectiveness of vitamin C for acute pain [12]. However, most studies included in that analysis [12] focused on other analgesics, with vitamin C being used as a placebo [13,14,15,16]. Another report investigated the analgesic effect of vitamin C in patients undergoing ocular surgeries through local administration and the results could not be extrapolated to other major surgeries and the administration of vitamin C through the systemic routes [17]. Therefore, the present meta-analysis attempted to shed light on the effectiveness of vitamin C against acute postoperative pain by systematically reviewing all the available clinical trials.

## 2. Materials and Methods

The present meta-analysis was conducted according to Preferred Reporting Items Systematic Reviews and Meta-Analysis (PRISMA) guidelines [18] and was registered on the International Prospective Register of Systematic Reviews (CRD42020202592).

### 2.1. Search Strategy

We searched the databases of Medline, Embase, Google Scholar, PubMed, and the Cochrane controlled trials register, and the U.S. National Library of Medicine clinical trial register (www.clinicaltrials.gov) to obtain a list of all published or unpublished eligible randomized controlled trials (RCTs) comparing the postoperative pain outcomes with or without perioperative use of vitamin C in patients requiring surgery using the keywords ”vitamin C”, “ascorbic acid”, “antioxidant”, pain”, “analgesia”, “opioid”, “morphine”, “pain score”, “anesthesia”, “postoperative”, “perioperative”, “surgery”, ”patient-controlled analgesia”, and “RCT” from inception to August 10, 2020. References from relevant studies were searched to find additional studies. No publication date or language restriction was applied.

### 2.2. Study Selection Criteria

Two reviewers (K.-H.C., Y.-T.L.) independently examined the abstracts of the acquired articles to identify potentially eligible studies. The PICO criteria for eligibility of randomized controlled trials for the current study included: (1) Population: adult surgical patients, age above or equal to 18 years old; (2) Intervention: vitamin C was given as an intervention rather than a control through oral or intravenous route; (3) Comparison: placebo or no therapy; and (4) Outcome: analgesic consumption and/or severity of pain within postoperative 48 h. There were no restrictions on dose or timing of administration. The exclusion criteria were (1) studies that focused on dental or ocular surgeries and/or pediatric population because of the relatively low severity of pain and the difficulty in pain assessment, respectively, (2) those in which information regarding dosage of vitamin C or acute pain outcomes was unavailable, and (3) those that adopted vitamin C as a placebo. Two authors (K.-C.H., J.-Y.C.) independently investigated the selected trials for the final analysis. If disagreements arose, a third author (C.-K.S.) was involved until a consensus was reached.

### 2.3. Data Extraction

Three authors (L.-K.W., S.-C.W., and J.-Y.C.) extracted relevant data from each selected trial and entered them into predefined databases. Divergences were resolved by discussion. If the included studies did not report data on primary or secondary outcomes, the corresponding authors were contacted for further information. The following data were extracted from each trial: author, publication year, study setting, patient characteristics, sample size, surgical procedure, dosage of vitamin C, blood loss, postoperative analgesic technique, postoperative opioid consumption, postoperative pain score (e.g., numerical rating scale (NRS)), and adverse events. All opioid consumption was converted into parenteral morphine equivalents (i.e., 0.1 mg parenteral fentanyl = 10 mg parenteral morphine; 75 mg parenteral meperidine = 10 mg parenteral morphine) [19].

### 2.4. Primary Outcome, Secondary Outcomes, and Definitions

Acute pain outcomes of the present meta-analysis were defined as the severity of pain or opioid consumption within postoperative 48 h. The primary endpoint was postoperative opioid consumption at postoperative 24 h, while the secondary outcomes included the severity of pain, postoperative opioid consumption at postoperative 1–2 h or at 48 h, postoperative circulating vitamin C concentration after vitamin C supplementation as well as the risks of postoperative nausea and vomiting (PONV). The severity of pain was defined according to the pain score of each study.

### 2.5. Assessment of Risk of Bias for Included Studies

Two authors (M.-H.C. and Y.-J.C.) assessed the risk of bias for each trial using the criteria outlined in the *Cochrane Handbook for Systematic Reviews of Interventions* [20]. Disagreements were solved by discussion. The overall risk of bias of all included studies and the risk of bias of individual studies were analyzed. We rated the potential risk of bias by applying a rating of “low”, “high,” or “unclear” to each trial.

### 2.6. Statistical Analysis

For dichotomous outcomes, a random effects model was used to calculate the risk ratios (RRs) with 95% confidence intervals (CIs). The Mantel–Haenszel (MH) method was used to pool dichotomous data and to compute pooled RRs with 95% CIs. For continuous outcome, the selected effect size was the weighted mean difference (WMD) for analgesic dosage or standardized mean difference (SMD) for pain severity. The WMD is a standard statistic that measures the absolute difference between the mean value in two groups, while SMD is used as a summary statistic when the studies all assess the same outcome but measure it in a variety of ways (e.g., comparison of pain severity using two different pain scores). We assessed the morphine-sparing effect of vitamin C by using the method proposed by a previous meta-analysis [21] that calculated the effect with the equation: Morphine-sparing effect = (WMD/median of the average cumulative morphine in placebo group) × 100% (where WMD was the reduction in morphine dosage in the study group). The *I*^2^ statistic was applied for heterogeneity assessment (low: 0% to 50%; moderate: 50% to 75%, high: 75% to 100%). Sources of heterogeneity were explored by prespecified subgroup analyses on the routes of administration (i.e., oral or intravenous). Sensitivity analyses were performed to explore the potential influence of a single trial on the overall findings by omitting the studies from the meta-analysis one at a time. We examined the funnel plots when we identified 10 or more studies reporting on a particular outcome to investigate the potentials of reporting and publication bias. The significance level was set at 0.05 for all analyses. Cochrane Review Manager (RevMan 5.4; Copenhagen: The Nordic Cochrane Center, The Cochrane Collaboration, 2014) was used for data synthesis.

## 3. Results

### 3.1. Study Selection

**Figure 1:**
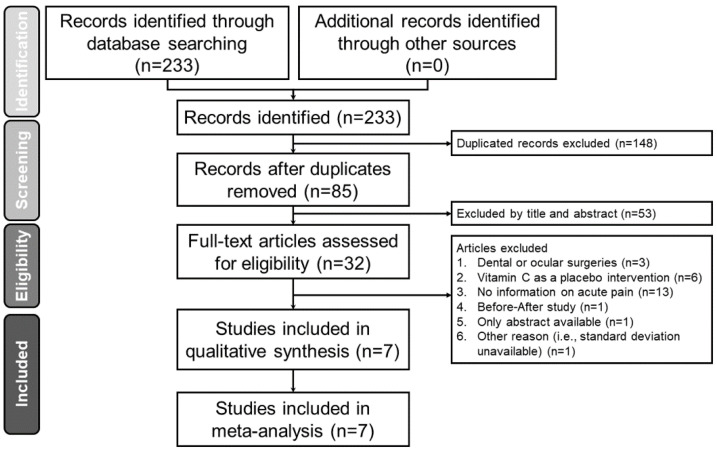
Preferred Reporting Items Systematic Reviews and Meta-Analysis (PRISMA) flowchart for selecting eligible studies.

Figure 1 is the Preferred Reporting Items for Systematic Reviews and Meta-Analyses (PRISMA) flow diagram that summarizes the reasons for study exclusion. Of a total of 227 potentially eligible reports retrieved from the database search, 146 were removed as they were duplicates. We then excluded 53 records after the initial review of the titles and abstracts. Of the 53 excluded studies, 39 did not mention vitamin C, seven did not involve surgery, five did not provide outcome on pain assessment, one focused on the pediatric population, and one was an animal study (Table S1). Overall, 28 studies were considered relevant and the full text was read. Another 21 articles were excluded because of the involvement of dental or ocular surgeries (n = 3), the use of vitamin C as a placebo intervention (n = 6), no information on acute pain outcomes (n = 13), a before-and-after study design (n = 1), availability of only an abstract (n = 1), and lack of data on standard deviation (n = 1). Finally, a total of seven randomized trials were included in the current meta-analysis (Figure 1).

### 3.2. Characteristics of Included Studies

Seven RCTs (n = 519 participants) published between 2012 to 2020 were analyzed. The study characteristics are described in Table 1. Vitamin C was given perioperatively in six trials [5,22,23,24,25,26], and was administered twice a day for three days in another trial [27]. The routes of administration included intravenous in five trials [5,22,23,26,27] and oral in two trials [24,25]. Patient-controlled analgesia (PCA) was used for postoperative pain control in five trials [23,24,25,26,27], while one study used boluses of analgesics [5] and the other [22] did not specify the intervention strategies. The follow-up time was 24 h in five trials [5,22,23,24,25], 48 h in one trial [26], and 72 h in the other trial [27]. All procedures were elective and most (i.e., five out of seven) were laparoscopic [22,23,24,26,27]. Two pain scores were adopted for pain severity assessment across the seven studies included, namely, the visual analog scale (VAS) [5,22,25] and the verbal numeric rating scale (NRS) [23,24,26,27]. The cumulative morphine consumption of the included studies at postoperative 24 h and the respective pain scores are summarized in Supplemental Table S2. Placebos used in the seven included studies were normal saline [5,22,23,26,27], carbonated orange beverage [24], and oral placebo tablet [25]. Dropout rates, which were mentioned in three studies [23,24,26], ranged from 2% to 3% in the vitamin group and from 4% to 9.1% in the placebo group.

### 3.3. Risk of Bias Assessment

**Figure 2:**
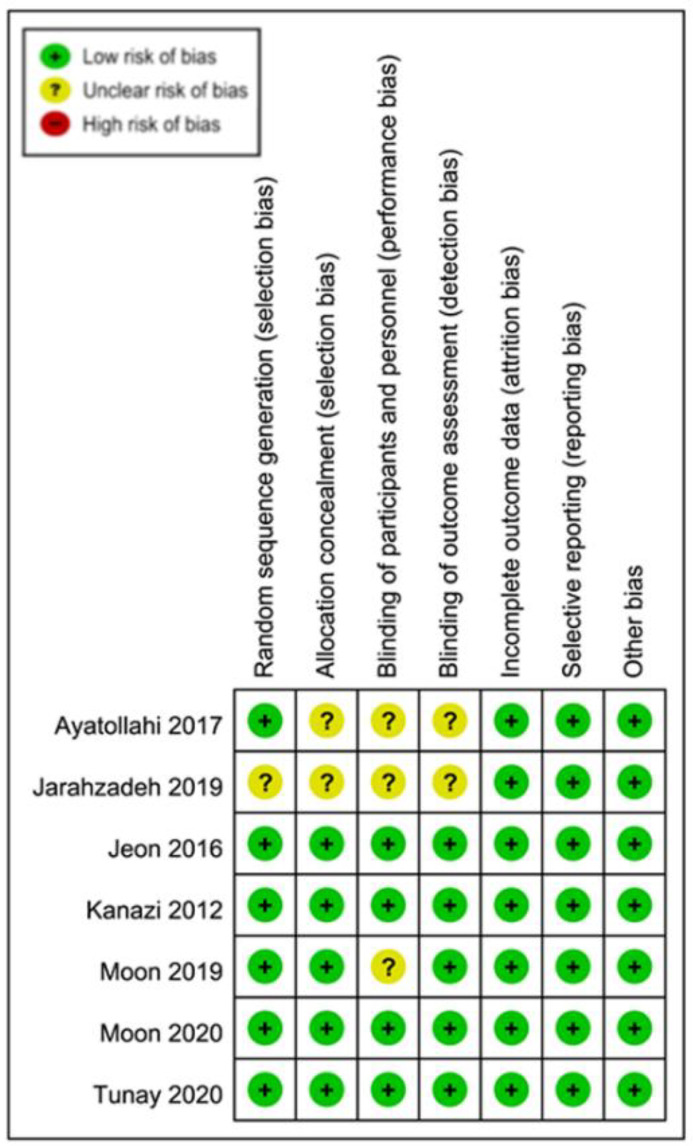
Risks of bias of individual studies.

**Figure 3:**
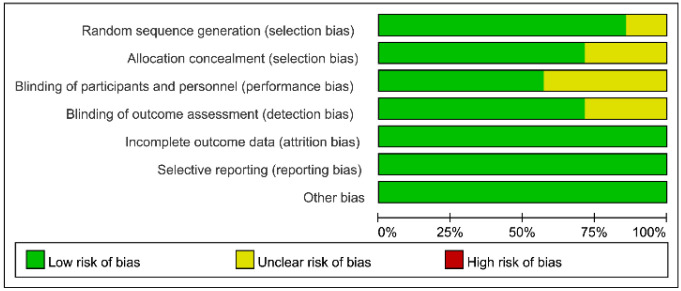
Overall risks of bias of the seven included studies.

The risks of bias of individual studies and the overall risk of bias are summarized in Figure 2 and Figure 3, respectively. Most included studies were found to give sufficient details about randomization and assigned a low risk of allocation bias [23,24,25,26,27]. Several studies were able to adopt methods to keep both the investigators unaware of the administration of vitamin C (e.g., the syringes were covered with black plastic sheets), the risk of performance bias of those trials was considered low [23,24,25,26]. Other risks of bias including attrition bias, measurement bias, reporting bias, and overall bias were also considered to be low in all studies. Detailed information on bias assessment of the included studies is shown in Supplemental Table S3.

### 3.4. Outcomes

#### 3.4.1. Severity of Pain within 1–2 h after Surgery

**Figure 4:**
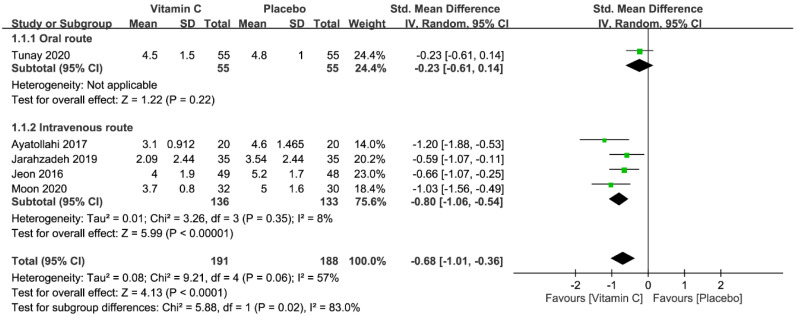
Forest plot for comparing the severity of pain within postoperative 1–2 h between vitamin and placebo groups. CI, confidence interval; IV, inverse variance; Std., standardized.

Five studies with a total of 379 patients (vitamin group, n = 191 vs. placebo group, n = 188) were available for the analysis of pain score within 1–2 h after surgery [5,22,23,25,26]. Pooled analysis showed a lower pain score in the vitamin group than that in the placebo group (SMD = −0.68, 95% CI −1.01 to −0.36, *p* < 0.0001; *I*^2^ = 57%) (Figure 4). In contrast to the overall result, subgroup analysis revealed no significant reduction in pain score among patients receiving vitamin C through the oral route (Figure 4). Sensitivity analysis demonstrated no significant impact on outcome by omitting certain trials.

#### 3.4.2. Severity of Pain 6 h after Surgery

**Figure 5:**
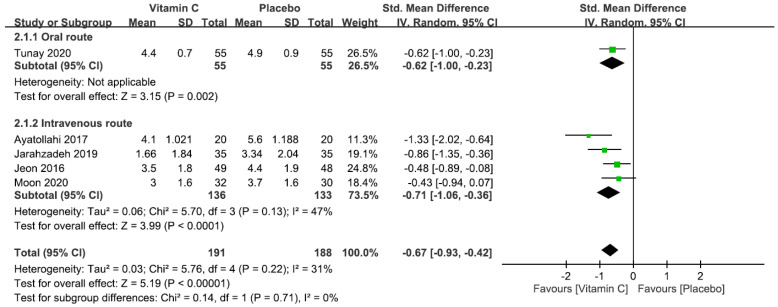
Forest plot for the comparison of pain severity 6 h after surgery between vitamin and placebo groups. CI, confidence interval; IV, inverse variance; Std., standardized.

Five studies with a total of 379 patients (vitamin group, n = 191 vs. placebo group, n = 188) were eligible for the analysis [5,22,23,25,26]. A forest plot, presented in Figure 5, demonstrated a lower mean pain score at postoperative 6 h in the vitamin group compared with that in the placebo group (SMD = −0.67, 95% CI −0.93 to −0.42, *p* < 0.00001; *I*^2^ = 31%) (Figure 5). Subgroup analysis based on the routes of administration selected (i.e., oral vs. intravenous) also showed consistent findings (Figure 5). Sensitivity analysis showed no significant impact on outcome by omitting certain trials.

#### 3.4.3. Severity of Pain at 24 h after Surgery

**Figure 6:**
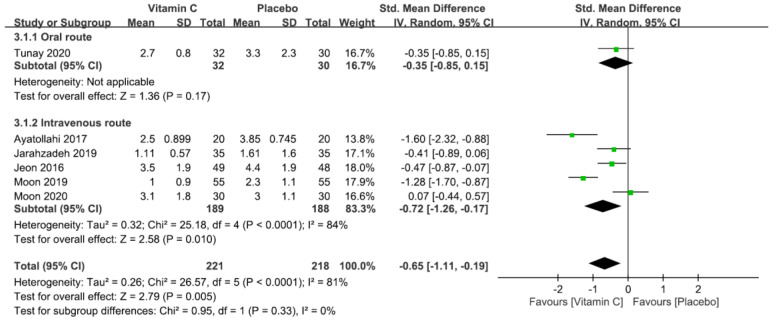
Forest plot for comparing the severity of pain at 24 h after surgery between vitamin and placebo groups. CI, confidence interval; IV, inverse variance; Std., standardized.

The forest plot on six available studies with a total of 439 patients (vitamin group, n = 221 vs. placebo group, n = 218) [5,22,23,25,26,27] (shown in Figure 6) demonstrated a lower pain severity at postoperative 24 h in the vitamin group compared with that in the placebo group (SMD = −0.65, 95% CI −1.11 to −0.19, *p* = 0.005; *I*^2^ = 81%) (Figure 6). Again, contrary to the overall result, subgroup analysis demonstrated that oral vitamin C supplementation was ineffective for pain alleviation (Figure 6) also showed similar findings. Sensitivity analysis found no significant impact on this outcome by omitting certain trials.

#### 3.4.4. Opioid Consumption within 1–2 h after Surgery

**Figure 7:**
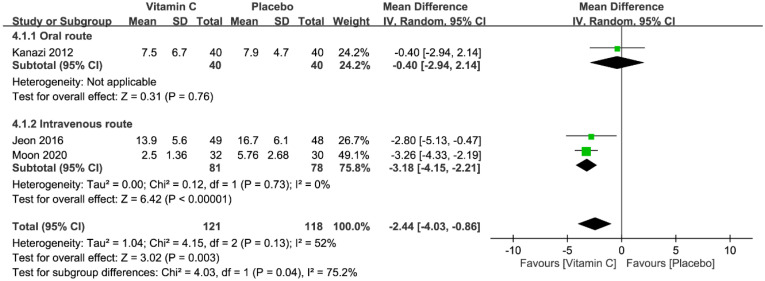
Forest plot for the comparison of opioid consumption (mg) within postoperative 1–2 h between vitamin and placebo groups. CI, confidence interval; IV, inverse variance; WMD, weighted mean difference.

Three studies with a total of 239 patients (vitamin group, n = 121 vs. placebo group, n = 118) were available for the analysis [23,24,26]. All studies used PCA for postoperative pain control. Within postoperative 1–2 h, the median of the average cumulative morphine dosages in the placebo groups was 7.9 mg (range, 5.76–16.7 mg). A forest plot, (presented in Figure 7) demonstrated a significantly lower morphine consumption in the vitamin group than that in the placebo group (WMD = −2.44 mg, 95% CI −4.03 to −0.86, p = 0.003; *I*^2^ = 52%), suggesting a vitamin C-associated morphine-sparing effect of 30.9% (i.e., 2.44 mg/7.9 mg × 100%). Nevertheless, subgroup analysis showed no significant reduction in opioid use when the oral route was chosen for vitamin C administration. Sensitivity analysis demonstrated no difference in morphine consumption between both groups when either the study by Jeon et al. [23] or that by Moon et al. [26] was omitted from the current meta-analysis.

#### 3.4.5. Opioid Consumption within 6 h after Surgery

**Figure 8:**
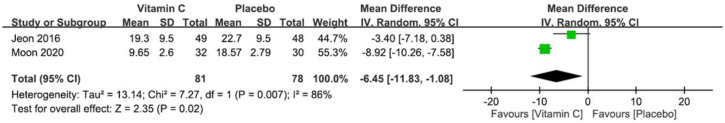
Forest plot for the comparing opioid consumption (mg) within 6 h after surgery between vitamin and placebo groups. CI, confidence interval; IV, inverse variance; WMD, weighted mean difference.

Two studies using PCA for postoperative pain control involving a total of 159 patients (vitamin group, n = 81 vs. placebo group, n = 78) were eligible for the analysis [23,26]. At 6 h, the median of the average cumulative morphine dosages in placebo groups was 20.6 mg (range, 18.57–22.7 mg). The forest plot demonstrated a lower morphine consumption in the vitamin group than that in the placebo group (WMD = −6.45 mg, 95% CI −11.83 to −1.08, *p* = 0.02; *I*^2^ = 86%) (Figure 8), showing a vitamin C-associated morphine-sparing effect of 31.3% (i.e., 6.45 mg/20.6 mg × 100%).

#### 3.4.6. Opioid Consumption within 24 h after Surgery

**Figure 9:**
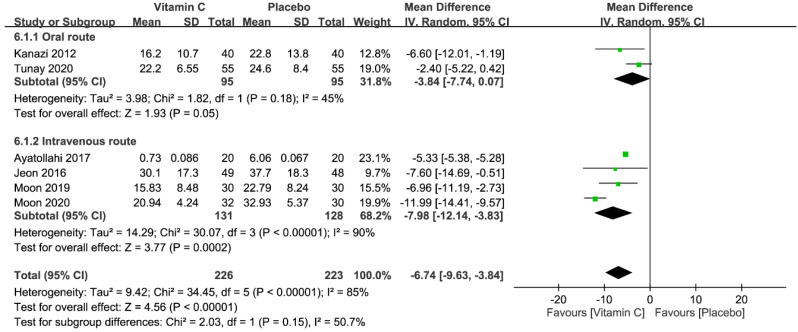
Forest plot for the comparison of opioid consumption (mg) within postoperative 24 h between vitamin and placebo groups. CI, confidence interval; IV, inverse variance.

Of the six studies with a total of 449 patients (vitamin group, n = 226 vs. placebo group, n = 223) available for the analysis [5,23,24,25,26,27], five used PCA for postoperative pain control [23,24,25,26,27]. At 24 h, the median of the average cumulative morphine dosages in the placebo groups was 23.7 mg (range, 6.06–37.7 mg). The forest plot demonstrated a lower morphine consumption in the vitamin group than that in the placebo group (WMD = −6.74 mg, 95% CI −9.63 to −3.84, *p* < 0.00001; *I*^2^ = 85%) (Figure 9), suggesting a vitamin C-associated morphine-sparing effect of 28.4% (i.e., 6.74 mg/23.7 mg × 100%). Despite the overall reduction in morphine use, subgroup analysis demonstrated that administration of vitamin C through the oral route only showed borderline significance in this aspect (*p* = 0.05). Sensitivity analysis demonstrated no significant impact on outcome by omitting certain trials.

#### 3.4.7. Opioid Consumption within 48 h after Surgery

**Figure 10:**
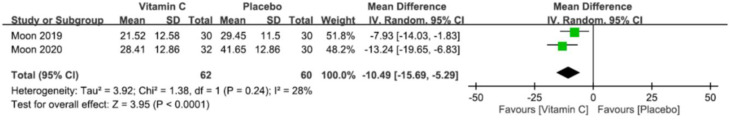
Forest plot for comparing opioid consumption (mg) within 48 h after surgery between vitamin and placebo groups. CI, confidence interval; IV, inverse variance.

Two studies utilizing PCA for postoperative pain control in a total of 122 patients (vitamin group, n = 62 vs. placebo group, n = 60) [26,27] were available for analysis, which showed a lower level of morphine consumption in the vitamin group than that in the placebo group (WMD = −10.49 mg, 95% CI −15.69 to −5.29, *p* < 0.0001; *I*^2^ = 28%) (Figure 10), suggesting a vitamin C-associated morphine-sparing effect of 29.5% (i.e., 10.49 mg/35.55 mg × 100%).

#### 3.4.8. Perioperative Blood Loss

**Figure 11:**
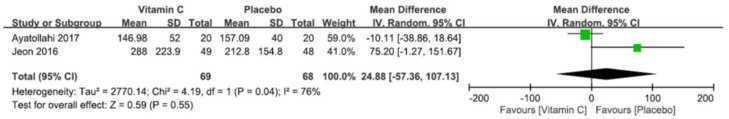
Forest plot for the comparison of perioperative blood loss between vitamin and placebo groups. CI, confidence interval; IV, inverse variance.

Two studies investigating the intraoperative blood loss in a total of 137 patients (vitamin group, n = 69 vs. placebo group, n = 68) [5,23] eligible for analysis demonstrated no significant difference in this outcome between the vitamin group and the placebo group (WMD = 24.88 mL, 95% CI −57.36 to 107.13, *p =* 0.55; *I*^2^ = 76%) (Figure 11).

#### 3.4.9. Postoperative Nausea and Vomiting

**Figure 12:**
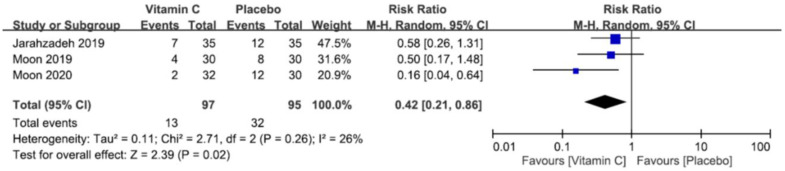
Forest plot for comparing the incidences of postoperative nausea or vomiting within postoperative 1–2 h between vitamin and placebo groups. CI, confidence interval; RR, risk ratio; M-H = Mantel–Haenszel.

**Figure 13:**
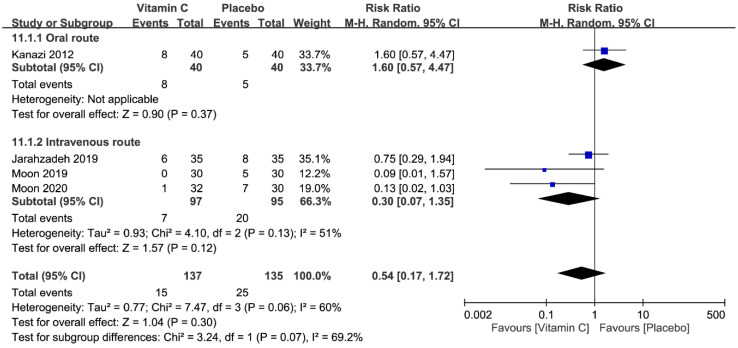
Forest plot for the comparison of the incidences of postoperative nausea or vomiting 24 h after surgery between vitamin and placebo groups. CI, confidence interval; RR, risk ratio; M-H = Mantel–Haenszel.

The pooled RRs of PONV at postoperative 1 h [22,26,27] and 24 h [22,24,26,27] were 0.42 (95% CI 0.21 to 0.86, *p* = 0.02, *I*^2^ = 26%) (Figure 12) and 0.54 (95% CI 0.17 to 1.72, *p* = 0.30, *I*^2^ = 60%) (Figure 13), respectively. The findings showed that the use of vitamin C was associated with a lower risk of PONV at postoperative 1 h. However, there was no significant difference in the risk of PONV between the two groups at postoperative 24 h.

#### 3.4.10. Postoperative Circulating Vitamin C Concentration after Vitamin C Supplementation

**Figure 14:**
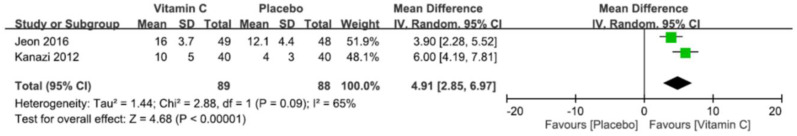
Forest plot for the comparison of postoperative circulating vitamin C concentration between vitamin and placebo groups. CI, confidence interval; IV, inverse variance; WMD, weighted mean difference.

Two studies investigating the postoperative vitamin C blood concentration in a total of 177 patients (vitamin group, n = 89 vs. placebo group, n = 88) [23,24] were available for analysis, which demonstrated a highly significant increase in circulating vitamin C concentration in patients with perioperative vitamin C supplementation compared with that in those without (WMD = 4.91 mg/L, 95% CI 2.85 to 6.97, *p <* 0.00001; *I*^2^ = 65%) (Figure 14).

## 4. Discussion

The current study, which is the first to systematically address the analgesic effectiveness of vitamin C against postoperative pain, had several striking clinical implications. First, our findings showed that vitamin C was associated with a reduced pain score at postoperative 1–2, 6, and 24 h as well as a decreased morphine requirement for up to postoperative 48 h. In addition, the incidence of postoperative nausea or vomiting at postoperative 1–2 h was reduced despite the lack of significant difference between the two groups at 24 h after surgery. Although a previous study reported that intraoperative administration of vitamin C was associated with a decreased operative blood loss [28], the present study demonstrated no significant difference in blood loss between the vitamin and placebo groups.

Postoperative pain control is an important concern because up to 75% of patients who experienced postoperative pain classified the severity of their pain as moderate, severe, or even extreme [29]. Even in the ambulatory surgery setting, a large-scale study on more than five thousand outpatients demonstrated significant pain sensation in up to 30% of the studied population with pain severity being moderate to severe [30]. Acute pain control is also crucial to the prevention of chronic postoperative pain [3,31]. A previous study has demonstrated a positive correlation between the intensity of acute postsurgical pain with the risk of persistent pain development [3]. Nevertheless, achieving a balance between postoperative pain management and minimization of analgesics-associated side-effects is always a challenge to clinicians. Therefore, despite the proven effectiveness of opioids and other common analgesics against postoperative pain, inadequate pain control was not uncommon because of their untoward side-effects [5]. Accordingly, modern clinical practice guidelines recommend a multimodal approach to analgesia, including the use of various analgesic medications and techniques in combination with non-pharmacological interventions [4]. One of the most common adjuvants to opioid-based analgesia are non-steroidal anti-inflammatory drugs (NSAIDs) which, however, are also associated with adverse side-effects [4,32,33,34]. Vitamin C, which is a water-soluble vitamin with the excesses in circulation rapidly excreted in the urine, has a remarkably low acute toxicity [35]. In this way, vitamin C may be a promising adjuvant to conventional analgesics.

To compare the morphine-sparing effect of vitamin C with that associated with other analgesics, we investigated previous meta-analytic studies that showed reductions in morphine requirement at 24 h by 14.7%, 20%, and 21% corresponding to 7.2, 9, and 10.3 mg for celecoxib (200 mg) [36], acetaminophen [37], and a single dose of NSAID [36], respectively. Therefore, a decrease by 28.4% in the current study appeared to be higher than that previously reported for other analgesics. As the above medications are popular clinical adjuvants to opioid-based regimens in the multi-modal approach to operative antinociception, the finding of the current study may imply the possibility of incorporating vitamin C into the postoperative care protocol.

Previous studies have attributed the antinociceptive effects of vitamin C to its antioxidative and neuromodulatory properties. A previous report has shown a positive association between reactive oxygen species (ROS) and neuropathic pain through the demonstration of pain alleviation by administering ROS scavengers to a rat model of single nerve ligation pain [38]. The analgesic action of vitamin C through its role as a free radical scavenger has been suggested in a previous experimental study [6]. With respect to neuromodulation, there is evidence linking the antinociceptive property of vitamin C to its action on the N-methyl-D-aspartate (NMDA) receptor. Previous investigations not only revealed that vitamin C could modulate the neurotransmission of glutamate and dopamine through altering the redox changes on the NMDA receptor [39] but also demonstrated that vitamin C exerted an antinociceptive effect in chemically induced animal pain models possibly through an inhibition of the ionotropic NMDA receptor [40]. Furthermore, vitamin C is critical for the biosynthesis of neurotransmitters known to be key components of the inhibitory pain pathway [41]. Being a substrate of the enzyme dopamine beta-hydroxylase for the conversion of dopamine to norepinephrine, vitamin C has an important role to play in this rate-limiting step in norepinephrine formation [42,43]. In addition, vitamin C participates in cholinergic and GABAergic transmission [44].

Clinically, because surgery is known to increase oxidative stress, which is associated with a reduction in postoperative plasma vitamin C level [45], a previous review has suggested an alleviation of the free radical burden among surgical patients through vitamin C supplementation to boost its circulating level [45]. This proposal is supported by two of our included studies that showed a significant elevation in plasma vitamin C level after a single bolus supplementation either through the intravenous route at a dose of 50 mg/kg [23] or orally at a dose of 2 g [24]. The antinociceptive effect of vitamin C was also evident in another study in which patients with post herpetic neuralgia, who were found to have a decreased plasma concentration of vitamin C, exhibited a reduction in spontaneous but not brush-evoked pain after the intravenous administration of vitamin C at 50 mg/kg with a maximum dose 2.5 g/day every other day for three doses [9]. An interesting finding of the current study was that despite the demonstration of a reduction in opioid dosage up to postoperative 48 h among patients with vitamin C administration compared to those without, our results showed no difference in the incidence of PONV between the two groups. The findings, therefore, suggested that PONV among the included patients may not be opioid related. Indeed, a previous meta-analysis [46] has shown that up to 50% to 75% of patients would experience PONV after laparoscopic procedures, which comprised five out of seven of our included studies. Taken together, the findings of the current meta-analysis supported the antinociceptive benefit of perioperative vitamin C supplementation without notable increase in side-effects among surgical patients [9,23,24].

Besides antinociception, another issue regarding patient safety is the association between vitamin C deficiency and surgical bleeding. Vitamin C is pivotal to platelet aggregation and prevention of platelet depletion in the process of hemostasis [47]. Spontaneous hemorrhage has been reported in patients with plasma vitamin C concentrations less than 0.6 mg/dL [48] due to impaired vascular integrity from defective collagen formation [48]. Therefore, vitamin C deficiency should be included as a differential diagnosis for surgical patients with nonspecific bleeding especially in those with severe illnesses, prolonged hospitalization, and poor dietary intake, which are known contributors to vitamin C deficiency [48]. Consistently, previous randomized controlled trials have demonstrated a significant reduction in surgical blood loss among patients undergoing abdominal myomectomy [28] and cardiopulmonary bypass surgery [47] with intraoperative vitamin C supplementation compared to those without. Nevertheless, the current study did not demonstrate a significant association between perioperative vitamin C supplementation and surgical blood loss.

One of the interesting findings of the present study was the differences in pain alleviation and morphine use between the intravenous and oral routes of vitamin C administration. While intravenous supplementation of vitamin C was found effective for decreasing pain severity at 1–2, 6, and 24 h after surgery, oral administration was associated with significant pain relief only at postoperative 6 h. Similarly, although intravenous vitamin C was significantly related to a reduction in morphine requirement immediately (1–2 h) and one day after surgery, similar therapeutic benefits were not seen for the oral route. Indeed, vitamin C administration through the intravenous route has been found to produce a 70-fold higher blood level than that when it was given orally at the maximum tolerable dose [49]. The downside of vitamin C administration through the oral route is the tightly controlled plasma concentration. In contrast, intravenous administration can attain a substantially higher blood concentration through bypassing such a tight control [49]. This may explain the enhanced effectiveness of intraoperative vitamin C administration through the intravenous route compared to that given orally in the current study.

The present study had its strengths and limitations. Because pain is a subjective feeling, analysis of morphine dosages from patient-controlled analgesia in five out of the seven included studies could reliably reflect the severity of pain for accurate assessment of the antinociceptive effect of vitamin C. In addition, we excluded patients with relatively low severity of postoperative pain including those undergoing dental and ocular surgeries as well as the pediatric population, who could not provide reliable information. Furthermore, the included studies for the present meta-analysis involved patients receiving similar procedures (i.e., laparoscopic surgeries in 5 out of 7 studies) so that variations in the severity of pain from different operations were minimized.

Nevertheless, the downside of the high homogeneity in surgical procedures also restricted the extrapolation of our findings to patients undergoing other operations. Another limitation was that the number of included studies was not large enough to analyze the publication bias. In addition, a single perioperative bolus of vitamin C in most studies (i.e., six out of seven) could not shed light on the possible additional benefits from repeated administrations for maintaining a high circulating level. Moreover, the follow-up periods of the included trials were too short (i.e., 24–48 h) to elucidate the long-term antinociceptive benefit of vitamin C as previous studies have demonstrated a reduction in chronic pain after perioperative vitamin C administration [50]. Moreover, statistically, although pooled analysis of the current study showed a lower pain score in the vitamin group compared to that in the placebo controls with substantial significance (i.e., *p* < 0.0001 within 1–2 h after surgery), the small *p* value only represented the unlikeliness that the observation happened by random chance [51] but did not actually reflect the efficacy of the intervention, which needs to be further investigated through large-scale clinical trials. Pharmacologically, because different analgesics were used in the included studies (e.g., fentanyl and meperidine), we used equianalgesic parenteral dosage conversion into parenteral morphine [19] for comparison. However, the accuracy of such conversions remains questionable because of a lack of professional consensus [19]. In the current study, we adopted the same conversion ratios for both vitamin and placebo groups to minimize the impact of such conversions on our outcomes. Furthermore, because the present meta-analysis aimed at investigating the impact of perioperative vitamin C administration on early postoperative opioid consumption, we did not use different keyword combinations such as “pediatric” and “vitamin C” OR “serum vitamin C concentration” and “pain” OR “long-term vitamin C supplementation” and “pain” OR “vitamin C” and “postoperative chronic pain” in our literature search so that the impacts of routine dietary vitamin C supplementation, circulating vitamin C concentration, and perioperative vitamin C administration on the incidence of chronic pain as well as the analgesic effect of vitamin C in the pediatric population remain unclear. Finally, the heterogeneity of the included studies including the differences in routes and dosages of vitamin C administration [52] may be potential a confounder of the present meta-analysis.

## 5. Conclusions

The results of this meta-analysis demonstrated significant postoperative reductions in opioid requirement as well as a decrease in pain severity in patients receiving perioperative vitamin C, suggesting that vitamin C may be incorporated into the multimodal approach to postoperative analgesia in surgical patients. Taking into consideration its effectiveness through intravenous administration and the low toxicity, further large-scale trials are warranted to elucidate its optimal intravenous dosage and antinociceptive role in other clinical settings.

## Figures and Tables

**Table 1:** Characteristics of the included studies.

Author/Year	Vitamin C Dosage	Time of Administration	Route	Analgesia Methods	Surgical Procedures	Surgical Time (V vs. P)	Patient Number /Age	Follow-Up Time
Ayatollahi 2017 [5]	3 g	30 min after the BOS	i.v.	Pethidine bolus ^‡^	Uvulopalatopharyngoplasty and tonsillectomy	111.8 ± 20.8 vs. 113.7 ± 20.9 min	n = 40; 25–50 years	24 h
Jarahzadeh 2019 [21]	2 g	30 min after the AI	i.v.	NA	Laparoscopic surgery	NA	n = 70; 20–60 years	24 h
Jeon 2016 [22]	50 mg/kg	Immediately after AI	i.v.	PCA with morphine	Laparoscopic colectomy	160.2 ± 39.3 vs. 160.7 ± 46.0 min	n = 97; 20–75 years	24 h
Kanazi 2012 [23]	2 g	60 min before AI	oral	PCA with morphine	Laparoscopic cholecystectomy	98.8 ± 33.6 vs. 91.3 ± 31.3 min	n = 80; 18–75 years	24 h
Tunay 2020 [24]	2 g	60 min before BOS	oral	PCA with morphine	Major abdominal surgery	84.5 ± 23.9 vs. 94.5 ± 27.5 min	n = 110, 18–65 years	24 h
Moon 2019 [26]	0.5 g twice a day	The day of surgery to the third day after surgery	i.v.	PCA with fentanyl	Laparoscopic hysterectomy	99.0 ± 30.3 vs. 96.0 ± 19.4 min	n = 60, 20–60 years	72 h
Moon 2020 [25]	50 mg/kg	Immediately prior to AI	i.v.	PCA with fentanyl	Laparoscopic gynecologic surgery	78.4 ± 34.7 vs. 92.8 ± 30 min	n = 66, 20–60 years	48 h

AI: anesthesia induction; BOS: beginning of surgery; i.v.: intravenous; PCA: patient-controlled analgesia; ^‡^ 1 g paracetamol for patients with pain score <5; pethidine 0.5 mg/kg for patients with pain score ≥5; V vs. P: vitamin C vs. placebo.

## Supplementary Materials

The following are available online at https://www.mdpi.com/2072-6643/12/10/3109/s1, Table S1: Reasons for exclusion of studies based on titles and abstracts, Table S2: Cumulative morphine consumptions at postoperative 24 h and the respective pain scores, Table S3: Risks of bias for included studies.
