# Supplementary material for: The Effect of Perioperative Vitamin C on Postoperative Analgesic Consumption: A Meta-Analysis of Randomized Controlled Trials
Source: Nutrients. 2020 Oct 12;12(10):3109. doi: 10.3390/nu12103109 (PMC7600013; doi:10.3390/nu12103109)
Supplement: Supplementary file 1 [file nutrients-12-03109-s001.pdf]

**Supplemental Table 1.** Reasons for exclusion of studies based on titles and abstracts (n=53)

| Reason for exclusion | Number of studies | Studies (Reference No.) |
|---|---|---|
| Vitamin C not mentioned | 39 | 1, 2, 3, 4, 5, 7, 8, 11, 12, 13, 14, 15, 17, 19, 20, 21, 24, 27, 28, 29, 30, 31, 32, 33, 34, 37, 38, 39, 40, 41, 42, 43, 44, 45, 46, 47, 50, 51, 52, |
| Surgery not involved | 7 | 9, 10, 16, 22, 35, 48, 49, |
| No outcome on pain assessment | 5 | 23, 25, 26, 36, 53 |
| Study on pediatric population | 1 | 18, |
| Animal study | 1 | 6, |

**References**


1. Tucker, A. T., Makings, E. & Benjamin, N. Study of a combined percutaneous local anaesthetic and nitric oxide-generating system for venepuncture. Anaesthesia 57, 429-433, doi:10.1046/j.0003-2409.2001.02394.x (2002).
2. Agarwal, B. B., Manish, K., Sahu, T., Sarangi, R. & Mahajan, K. C. Do dietary spices impair the patient reported outcomes in stapled hemorrhoidopexy ? A randomized controlled study. *Surgical endoscopy and other interventional techniques* **24**, S210-S211, doi:10.1007/s00464-010-0978-1 (2010).
3. Agarwal, K. A., Tripathi, C. D., Agarwal, B. B. & Saluja, S. Efficacy of turmeric (curcumin) in pain and postoperative fatigue after laparoscopic cholecystectomy: A double-blind, randomized placebo-controlled study. *Surgical Endoscopy* **25**, 3805-3810, doi:10.1007/s00464-011-1793-z (2011).
4. Wang, X. H. et al. Auricular acupoint pressing therapy in the treatment of obstructive sleep apnea syndrome. *Chinese journal of clinical rehabilitation* **10**, 165-167 (2006)
5. Alshiek, J. A. *et al.* Anti-oxidative treatment with vitamin E improves peripheral vascular function in patients with diabetes mellitus and Haptoglobin 2-2 genotype: A double-blinded cross-over study. *Diabetes research and clinical practice* **131**, 200-207, oi:10.1016/j.diabres.2017.06.026 (2017).
6. Arcos, M., Palanca, J. M., Montes, F. & Barrios, C. Antioxidants and gabapentin prevent heat hypersensitivity in a neuropathic pain model. *Journal of Investigative Surgery* **26**, 109-117, doi:10.3109/08941939.2012.713444 (2013).
7. Aydogan, N. H. *et al.* The effect of arthroscopic surgery and intraarticular drug injection to the antioxidation system and lipid peroxidation at osteoarthritis of knee. *Saudi medical journal* **29**, 397-402 (2008).

8. Bagger-Sjoback, D. *et al.* A randomised, double blind trial of N-Acetylcysteine for hearing protection during stapes surgery. *PloS one* **10**, e0115657, doi:10.1371/journal.pone.0115657 (2015).
9. Baillie, J. K. *et al.* Oral antioxidant supplementation does not prevent acute mountain sickness: double blind, randomized placebo-controlled trial. *QJM : monthly journal of the Association of Physicians* **102**, 341-348, doi:10.1093/qjmed/hcp026 (2009).
10. Balius, R. *et al.* A 3-Arm Randomized Trial for Achilles Tendinopathy: Eccentric Training, Eccentric Training Plus a Dietary Supplement Containing Mucopolysaccharides, or Passive Stretching Plus a Dietary Supplement Containing Mucopolysaccharides. *Current Therapeutic Research - Clinical and Experimental* **78**, 1-7, doi:10.1016/j.curtheres.2016.11.001 (2016).
11. Behboodi Moghadam, Z., Amirsalari, S. & Rezai, E. Effect of garlic on dysmenorrhea in women with endometriosis. *International Journal of Fertility and Sterility* **10**, 111, doi:10.22074/ijfs.2016.5054 (2016).
12. Bektas, S. G. *et al.* Does high thoracic epidural analgesia with levobupivacaine preserve myocardium? A prospective randomized study. *Biomed research international* **2015**, 658678, doi:10.1155/2015/658678 (2015).
13. Bonetta, A., Derelli, R. & Di Pierro, F. Cranberry extracts reduce urinary tract infections during radiotherapy for prostate adenocarcinoma. *Anticancer research* **31**, 1849-1850 (2011).
14. Bruhn, J. Pain rebound in day surgery: how can we avoid it? *Regional anesthesia and pain medicine* **44**, A8-A10, doi:10.1136/rapm-2019-ESRAABS2019.6 (2019).
15. Byval'tsev, V. A., Vikulina, E. P., Titova, N. M. & Sorokovikov, V. A. Amplification of therapeutic effect after inclusion of hyperbaric oxygenation into complex conservative management of patients with recurrent pain syndrome following microdiskectomy. *Zhurnal voprosy neirokhirurgii imeni n n burdenko*, 30-35 (2008).
16. Campos, V., Steiner, D., Santos, K. D. & Capucho, L. A prospective split-face double-blind randomized placebo-controlled trial to assess the efficacy of vitamin C and ferulic acid serum postfractional ablative laser for skin rejuvenation. *Journal of the american academy of dermatology.* **70**, AB13, doi:10.1016/j.jaad.2014.01.054 (2014).
17. Caumo, W. *et al.* The clinical impact of preoperative melatonin on postoperative outcomes in patients undergoing abdominal hysterectomy. *Anesthesia and analgesia* **105**, 1263-1271, table of contents, doi:10.1213/01.ane.0000282834.78456.90 (2007).
18. Charlton, A. J., Harvey, B. A., Hatch, D. J. & Soothill, J. F. Neutrophil mobility during anaesthesia in children. A trial of ascorbate premedication. *Acta anaesthesiologica Scandinavica* **31**, 343-346, doi:10.1111/j.1399-6576.1987.tb02580.x (1987).
19. Chen, X. *et al.* Dissociation between urate and blood pressure in mice and in people with early Parkinson's disease. *EBioMedicine* **37**, 259-268, doi:10.1016/j.ebiom.2018.10.039

(2018).
20. Chotton, T., Singh, N. R., Singh, L. C., Laithangbam, P. S. & Singh, H. S. The effect of pregabalin for relief of postoperative pain after abdominal hysterectomy. *JMS - Journal of Medical Society* **28**, 18-21, doi:10.4103/0972-4958.135219 (2015).
21. Cui, Y. *et al.* Efficacy of a self-management program in patients with chronic viral hepatitis in China. *BMC nursing* **18**, N.PAG, doi:10.1186/s12912-019-0366-7 (2019).
22. de Paula, E. A., Kossatz, S., Fernandes, D., Loguercio, A. D. & Reis, A. Administration of ascorbic acid to prevent bleaching-induced tooth sensitivity: a randomized triple-blind clinical trial. *Operative dentistry* **39**, 128-135, doi:10.2341/12-483-C (2014).
23. Djoric, P. *et al.* Distal venous arterialization and reperfusion injury: focus on oxidative status. *European surgical research. Europaische chirurgische forschung. Recherches chirurgicales europeennes* **48**, 200-207, doi:10.1159/000338619 (2012).
24. Dugieva, M. Z., Kotenko, K. V. & Morozova, K. V. Impact of hypoxen therapy on postoperative course in gynecologic patients. *Antibiotiki i khimioterapiia = antibiotics and chemoterapy [sic]* **57**, 28-31 (2012).
25. East-Powell, M. & Reid, R. Medical synopsis: Antioxidant supplementation may support reduction in pelvic pain in endometriosis. *Advances in Integrative Medicine* **6**, 181-182, doi:10.1016/j.aimed.2019.07.004 (2019).
26. Ener, K. *et al.* Evaluation of oxidative stress status and antioxidant capacity in patients with painful bladder syndrome/interstitial cystitis: preliminary results of a randomised study. *International urology and nephrology* **47**, 1297-1302, doi:10.1007/s11255-015-1021-1 (2015).
27. Femiano, F. & Scully, C. Burning mouth syndrome (BMS): double blind controlled study of alpha-lipoic acid (thioctic acid) therapy. *Journal of oral pathology & medicine : official publication of the International Association of Oral Pathologists and the American Academy of Oral Pathology* **31**, 267-269, doi:10.1034/j.1600-0714.2002.310503.x (2002).
28. Fentiman, I. S., Caleffi, M. & Tutt, P. Consequences of the administration of an anti-oestrogen to women with mastalgia and breast cancer. *Reviews on endocrine-related cancer* **Suppl 20**, 25-28 (1987).
29. Gach, J. E., Humphreys, F. & Berth-Jones, J. Randomized, double-blind, placebo-controlled pilot study to assess the value of free radical scavengers in reducing inflammation induced by cryotherapy. *Clinical and experimental dermatology* **30**, 14-16, doi:10.1111/j.1365-2230.2004.01694.x (2005).
30. Gautier, A. *et al.* Effects of Ovariohysterectomy and Hyperbaric Oxygen Therapy on Systemic Inflammation and Oxidation in Dogs. *Frontiers in Veterinary Science* **6**, doi:10.3389/fvets.2019.00506 (2020).
31. Gocmen, G., Gonul, O., Oktay, N. S., Yarat, A. & Goker, K. The antioxidant and anti-inflammatory efficiency of hyaluronic acid after third molar extraction. *Journal of Cranio-*

*Maxillofacial Surgery* **43**, 1033-1037, doi:10.1016/j.jcms.2015.04.022 (2015).

32. Gogas, H. J. *et al.* Adverse events associated with encorafenib plus binimetinib in the COLUMBUS study: incidence, course and management. *European journal of cancer* **119**, 97-106, doi:10.1016/j.ejca.2019.07.016 (2019).
33. Gokakin, A. K., Sancakdar, E., Atabey, M. & Topcu, O. Oxidative stress markers in laparoscopic versus open appendectomy for uncomplicated acute appendicitis: a double-blind randomized study. *Surgical endoscopy and other interventional techniques.* **28**, S181, doi:10.1007/s00464-014-3484-z (2014).
34. Gorecki, P. *et al.* Perioperative supplementation with a fruit and vegetable juice powder concentrate and postsurgical morbidity: A double-blind, randomised, placebo-controlled clinical trial. *Clinical Nutrition* **37**, 1448-1455, doi:10.1016/j.clnu.2017.08.004 (2018).
35. Goyal, A. & Mansel, R. E. A randomized multicenter study of gamolenic acid (Efamast) with and without antioxidant vitamins and minerals in the management of mastalgia. *The breast journal* **11**, 41-47, doi:10.1111/j.1075-122X.2005.21492.x (2005).
36. Gupte, S. R. & Savant, N. S. Post suxamethonium pains and vitamin C. *Anaesthesia* **26**, 436-440, doi:10.1111/j.1365-2044.1971.tb04818.x (1971).
37. Hamamsy, M. E., Bondok, R., Shaheen, S. & Eladly, G. H. Safety and efficacy of adding intravenous N-acetylcysteine to parenteral L-alanyl-L-glutamine in hospitalized patients undergoing surgery of the colon: a randomized controlled trial. *Annals of Saudi medicine* **39**, 251-257, doi:10.5144/0256-4947.2019.251 (2019).
38. Han, Y. *et al.* Differential efficacy of methylcobalamin and alpha-lipoic acid treatment on symptoms of diabetic peripheral neuropathy. *Minerva endocrinologica* **43**, 11-18, doi:10.23736/s0391-1977.16.02505-0 (2018).
39. Herr, G. E. G. *et al.* Effects of the use of bioceramic wraps in patients with lower limb venous ulcers: A randomized double-blind placebo-controlled trial. *Journal of Integrative Medicine* **18**, 26-34, doi:10.1016/j.joim.2019.11.006 (2020).
40. Hillier, S. L. *et al.* Phase 2, Randomized, Control Trial of Group B Streptococcus (GBS) Type III Capsular Polysaccharide-tetanus Toxoid (GBS III-TT) Vaccine to Prevent Vaginal Colonization With GBS III. *Clinical infectious diseases* **68**, 2079-2086, doi:10.1093/cid/ciy838 (2019).
41. Hsin-Ti, L. *et al.* The Effect in Topical Use of Lycogen(TM) via Sonophoresis for Anti-aging on Facial Skin. *Current pharmaceutical biotechnology* **16**, 1063-1069, doi:10.2174/1389201016666150731112010 (2015).
42. Hu, Y. *et al.* Effect of laparoscopic myomectomy on serum levels of IL-6 and TAC, and ovarian function. *Experimental and therapeutic medicine* **18**, 3588-3594, doi:10.3892/etm.2019.7941 (2019).
43. Ivry, M., David, G., Wiam, W. & Haim, B. Melatonin premedication improves quality of recovery following bariatric surgery – a double blind placebo controlled prospective

study. *Surgery for Obesity and Related Diseases* **13**, 502-506, doi:10.1016/j.soard.2016.11.001 (2017).

44. Jørgensen, H. B. *et al.* The effect of leucocyte platelet rich plasma (L-PRP) administrered in the wound on healing after hip arthroplasty. *Clinical Nutrition* **35**, S239 (2016).
45. Joseph, T. T., Krishna, H. M. & Kamath, S. Premedication with gabapentin, Alprazolam or a placebo for abdominal hysterectomy: Effect on preoperative anxiety, Postoperative pain and morphine consumption. *Indian Journal of Anaesthesia* **58**, 693-699, doi:10.4103/0019-5049.147134 (2014).
46. Ziegeler, S. et al. Effects of haemofiltration and mannitol treatment on cardiopulmonary-bypass induced immunosuppression. *Scand J Immunol* **69**, 234-241, doi:10.1111/j.1365-3083.2008.02216.x (2009).
47. Kılıç, E. & Uğur, M. Effect of therapeutic hypothermia on superficial surgical site infection and postoperative pain in urgent abdominal surgery. *Turkish journal of trauma & emergency surgery / ulusal travma ve acil cerrahi dergisi* **24**, 417-422, doi:10.5505/tjtes.2018.23345 (2018).
48. Kim, B. *et al.* Comparative Evaluation of the Efficacy of Polyethylene Glycol With Ascorbic Acid and an Oral Sulfate Solution in a Split Method for Bowel Preparation: A Randomized, Multicenter Phase III Clinical Trial. *Diseases of the colon and rectum* **60**, 426-432, doi:10.1097/dcr.0000000000000759 (2017).
49. Kirk, G. R. *et al.* Combined Antioxidant Therapy Reduces Pain and Improves Quality of Life in Chronic Pancreatitis. *Journal of Gastrointestinal Surgery* **10**, 499-503, doi:10.1016/j.gassur.2005.08.035 (2006).
50. Kocamer, B. *et al.* Effects of caudal analgesia on oxidative stress response in paediatric cardiac surgery. *Applied cardiopulmonary pathophysiology.* **16**, 201-202 (2012).
51. Kostopanagiotou, G. *et al.* Effects of mannitol in the prevention of lipid peroxidation during liver resection with hepatic vascular exclusion. *Journal of clinical anesthesia* **18**, 570-574, doi:10.1016/j.jclinane.2006.03.014 (2006).
52. Lee, B. *et al.* Effect of ulinastatin on postoperative renal function in patients undergoing robot-assisted laparoscopic partial nephrectomy: a randomized trial. *Surg Endosc* **31**, 3728-3736, doi:10.1007/s00464-017-5608-8 (2017).
53. Lee, J. Y., Kim, C. J. & Chung, M. Y. Effect of high-dose vitamin C on oxygen free radical production and myocardial enzyme after tourniquet ischaemia-reperfusion injury during bilateral total knee replacement. *The Journal of international medical research* **38**, 1519-1529, doi:10.1177/147323001003800436 (2010).

**Supplemental Table 2.** Cumulative morphine consumptions at postoperative 24 hour and the respective pain scores

| | Vitamin C group | | Placebo group | |
|---|---|---|---|---|
| Study | Morphine (mg) | Pain scale | Morphine (mg) | Pain scale |
| Ayatollahi 2017[5] | 0.73 ± 0.086 | 2.5 ± 0.899‡ | 6.06 ± 0.067 | 3.85 ± 0.745‡ |
| Jarahzadeh 2019[22] | NA | 1.11 ± 0.57‡ | NA | 1.61 ± 1.6‡ |
| Jeon 2016[23] | 30.1 ± 17.3 | 3.5 ± 1.9¶ | 37.7 ± 18.3 | 4.4 ± 1.9¶ |
| Kanazi 2012[24] | 16.2 ± 10.7 | NA | 22.8 ± 13.8 | NA |
| Moon 2019[27] | 15.83 ± 8.48 | 1 ± 0.9¶ | 22.79 ± 8.24 | 2.3 ± 1.1¶ |
| Moon 2020[26] | 20.94 ± 4.24 | 3.1 ± 1.8¶ | 32.93 ± 5.37 | 3.0 ± 1.1¶ |
| Tunay 2020[25] | 22.2 ± 6.55 | 2.7 ± 0.8‡ | 24.6 ± 8.4 | 3.3 ± 2.3‡ |

‡visual analog scale; ¶verbal numeric rating scale; NA: not available

**Supplemental Table 3.** Risks of bias for included studies

*Ayatollahi 2017[5]*

| Bias | Authors' judgment | Support for judgment |
|---|---|---|
| Random sequence generation | Low risk | Each patient was assigned to one of the two groups according to a random number table. |
| Allocation concealment | Unclear risk | No descriptions on allocation concealment |
| Performance bias | Unclear risk | To ensure appropriate blinding of the rest of the team, group C (vitamin C) received infusion of 3 g of vitamin C in 500 mL of Ringer and group P (placebo) received 6 mL normal saline in 500 mL of Ringer. |
| Detection bias | Unclear risk | The information regarding blinding of outcome assessment was not described. |
| Attrition bias | Low risk | No patient lost on follow-up in both groups. |
| Reporting bias | Low risk | Main outcomes were measured and analyzed in accordance with a prespecified plan. |
| Other bias | Low risk | There was no conflict of interest. |

*Jarahzadeh 2019[22]*

| Bias | Authors' judgment | Support for judgment |
|---|---|---|
| Random sequence generation | Unclear risk | No description on random sequence generation. |
| Allocation concealment | Unclear risk | No description on allocation concealment. |
| Performance bias | Unclear risk | The experimental group received vitamin C (2 g) mixed with normal saline for a total injection volume of 500 mL during 30 minutes and those in the control group received normal saline 500 mL without vitamin C. |
| Detection bias | Unclear risk | The information regarding blinding of outcome assessment was not described. |

| | | |
|---|---|---|
| Attrition bias | Low risk | No patient lost on follow-up in both groups. |
| Reporting bias | Low risk | Main outcomes were measured and analyzed in accordance with a prespecified plan. |
| Other bias | Low risk | There was no conflict of interest. |

***Jeon 2016[23]***

| Bias | Authors' judgment | Support for judgment |
|---|---|---|
| Random sequence generation | Low risk | Patients were randomly allocated to two groups using a computer-generated randomization table. |
| Allocation concealment | Low risk | Group allocation was concealed in sealed opaque envelopes. |
| Performance bias | Low risk | Immediately after induction of anesthesia, a nurse who played no other role in the study selected an envelope for each patient and prepared an injection according to the group allocation. Patients in the vitamin C group received vitamin C 50mg/kg mixed with normal saline for a total injection volume of 50 mL, and those in the placebo group received normal saline 50 mL. The syringes were covered with black plastic and the solution was infused over 30 min using an infusion pump. |
| Detection bias | Low risk | A research assistant who was blinded to the study group assignments checked patients for pain, fatigue, postoperative nausea and vomiting (PONV), morphine consumption, and rescue analgesic requirement every 10 min during their stay in the post-anesthesia care unit (PACU) and again at 2, 6, and 24 h after discharge from the PACU to the ward. |
| Attrition bias | Low risk | Three patients were excluded from the final analysis due to complications. The withdraw rate was low and acceptable. The reasons of lost on follow-up were mentioned. |
| Reporting bias | Low risk | Main outcomes were measured and analyzed in accordance with a prespecified plan. |
| Other bias | Low risk | There was no conflict of interest. |

*Kanazi 2012[24]*

| Bias | Authors' judgment | Support for judgment |
|---|---|---|
| Random sequence generation | Low risk | Eighty-four patients were randomized using a computer-generated randomization table that was produced by the research assistant |
| Allocation concealment | Low risk | Group allocation was concealed in sealed opaque envelopes that were numbered and opened sequentially after patient consent had been obtained. |
| Performance bias | Low risk | Patients were then assigned to receive either 2 g of vitamin C or a placebo. Vitamin C effervescent tablets were dissolved in 15 mL of water. An equivalent volume of carbonated orange beverage having exactly the same color and taste was used for placebo. |
| Detection bias | Low risk | All patients were evaluated for pain, sedation, nausea, vomiting, pruritus, morphine consumption, and vital signs every 15 min during their stay in the PACU and at two, four, eight, 12, 18, and 24 hr after surgery. These evaluations were performed by a blinded anesthesiologist who was not involved in the study; preoperative staff, operating room staff, recovery room staff, ward nurses, and statisticians were also blinded. |
| Attrition bias | Low risk | Four patients were further excluded for protocol violation (wound infiltration with bupivacaine), one patient from the vitamin C group and three patients from the placebo group. The withdraw rate was low and acceptable. The reasons of lost on follow-up were mentioned. |
| Reporting bias | Low risk | Main outcomes were measured and analyzed in accordance with a prespecified plan. |
| Other bias | Low risk | There was no conflict of interest. |

*Moon 2019[27]*

| Bias | Authors' judgment | Support for judgment |
|---|---|---|
| Random sequence generation | Low risk | Patients were randomly assigned to one of the two groups according to the sealed envelope system. |

| | | |
|---|---|---|
| Allocation concealment | Low risk | Patients were randomly assigned to one of the two groups according to the sealed envelope system. |
| Performance bias | Unclear risk | The vitamin C group (Group C, n = 30) received 500 mg of vitamin C in 50 ml isotonic saline infusion intravenously over 10 min twice a day. The saline group (Group S, n = 30) received the same volume of isotonic saline over the same period. The infusion was prepared by personnel at the pharmacy and administered by the nurse (or anesthesiologist) who was blinded to the patient's group assignment. |
| Detection bias | Low risk | The data were subsequently collected and analyzed by an anesthesiologist who was also blinded to the study groups. |
| Attrition bias | Low risk | No patient lost on follow-up in both groups. |
| Reporting bias | Low risk | Main outcomes were measured and analyzed in accordance with a prespecified plan. |
| Other bias | Low risk | There was no conflict of interest. |

***Moon 2020[26]***

| Bias | Authors' judgment | Support for judgment |
|---|---|---|
| Random sequence generation | Low risk | Patients were randomly allocated using sealed envelope method. |
| Allocation concealment | Low risk | Study drugs were prepared by a pharmacist equipped only with a random assignment table. |
| Performance bias | Low risk | Vitamin C group (Group V, n = 33): 40 ml of a solution of 50 mg/kg of vitamin C (Ascorbic Acid Injection®, Huons Co., Korea) and isotonic saline is injected at a rate of 120 ml/h for 20 min as a bolus immediately prior to anesthesia induction. Subsequently, 40 ml of isotonic saline is infused at a rate of 10 mL/hr by continuous intravenous infusion until the end of the operation. Control group (Group C, n = 33): 40 ml of isotonic saline is injected at a rate of 120 mL/hr for 20 min as a bolus immediately prior to anesthesia induction. Subsequently, 40 mL of isotonic saline was infused at a rate of 10 mL/hr by continuous intravenous |

| | | |
|---|---|---|
| | | infusion until the end of the operation. Owing to the yellow color of vitamin C, all syringes were wrapped in aluminum foil. |
| Detection bias | Low risk | Data were collected by an anesthesiologist who was blinded to the study groups |
| Attrition bias | Low risk | Three patient lost follow-up in control group, and one patient lost follow-up in vitamin C group. The withdraw rate was low and acceptable. |
| Reporting bias | Low risk | Main outcomes were measured and analyzed in accordance with a prespecified plan. |
| Other bias | Low risk | There was no conflict of interest. |

***Tunay 2020[25]***

| Bias | Authors' judgment | Support for judgment |
|---|---|---|
| Random sequence generation | Low risk | The patients were randomly allocated to one of two groups of 55, each using a computer-generated random number assignment. |
| Allocation concealment | Low risk | After assignment to interventions, the trial participants, care providers, and outcome assessors were all blinded. |
| Performance bias | Low risk | Patients in group C (n = 55) received 2 g of vitamin C (Solgar Vitamin C 1000 mg tb), and patients in group P (n = 55) received a placebo tablet, orally, in the preoperative unit. The tablets were given by an anesthetist who was not one of the observers, to ensure the double-blind design. |
| Detection bias | Low risk | After assignment to interventions, the trial participants, care providers, and outcome assessors were all blinded. |
| Attrition bias | Low risk | There was no participant who discontinued or deviated from intervention protocols. |
| Reporting bias | Low risk | Main outcomes were measured and analyzed in accordance with a prespecified plan. |
| Other bias | Low risk | There was no conflict of interest. |
